# Marriage, Dependent Care, and Burnout Among Medical Students

**DOI:** 10.1001/jamanetworkopen.2025.59889

**Published:** 2026-02-16

**Authors:** Mytien Nguyen, Sarwat I. Chaudhry, Alexandra M. Hajduk, Tonya L. Fancher, Shruthi Venkataraman, Dowin Boatright

**Affiliations:** 1 Department of Immunobiology, Yale School of Medicine, New Haven, Connecticut; 2 Section of General Internal Medicine, Department of Medicine, Yale School of Medicine, New Haven, Connecticut; 3 Section of Geriatrics, Department of Internal Medicine, Yale School of Medicine, New Haven, Connecticut; 4 Division of General Internal Medicine, University of California Davis, School of Medicine, Sacramento; 5 Department of Emergency Medicine, New York University Grossman School of Medicine, New York

## Abstract

This cross-sectional study examines the intersection of marriage, dependent care, and burnout among US medical students.

## Introduction

Burnout among medical students affects future health care professionals’ well-being and retention.^1,2^ Examining how marriage and dependent care are associated with burnout is critical, as these factors may shape students’ experiences in a meaningful way. While existing research indicates that female sex, marital status, and having dependents are associated with lower burnout risk,^3^ the intersection between these characteristics and burnout in medical students remains underexplored.

## Methods

This cross-sectional study used fully deidentified data and was exempt from review per criteria from the New York University Langone Health institutional review board. We conducted a cross-sectional study of Association of American Medical Colleges 2017 to 2024 Graduation Questionnaire (GQ) respondents who matriculated between 2013 and 2021. Demographic variables included self-reported sex from the American Medical College Application Service and marital and dependent status from the GQ. Exhaustion and disengagement was assessed using the 16-item Oldenburg Burnout Inventory for Medical Students. Students scoring in the top quartile were classified as at risk for exhaustion (scores: 16 to 24) and disengagement (scores: 11 to 24), as previously described.^2,4^ Classifying burnout at the top-quartile enhances clinical interpretability by translating scores into a high-risk group, aligning with how wellness programs identify students needing intervention.

Modified Poisson regression was used to estimate the adjusted relative risk (aRR) of burnout by marital and dependent status, sex, and their intersections, clustering by medical school. Students with missing data were excluded from analysis. Interactions were assessed in separate models. Significance was determined using 2-sided *P* <.05. Analyses were performed using Stata 18.0 (StataCorp) and followed the STROBE guideline.

## Results

Among 155 709 graduates, 127 092 GQ (81.6%) responded to the GQ; 114 016 (89.7%) of whom had complete demographic information. Among 114 016 students, 59 805 (52.5%) identified as female. The prevalence of high-risk exhaustion and disengagement was 23.9% (27 268 students) and 23.0% (26 292 students) with mean (SD) scores of 8.2 (3.5) and 12.7 (4.2), respectively. Compared with single students, married students had a lower risk of exhaustion (aRR, 0.81; 95% CI, 0.79-0.84) and disengagement (aRR, 0.91; 95% CI, 0.88-0.93). Students with dependents had lower risks of exhaustion (aRR, 0.79; 95% CI, 0.75-0.83) and disengagement (aRR, 0.93; 95% CI, 0.90-0.98) compared with those without dependents (Table 1).

**Table 1.** Association of Marital and Dependent Status With Burnout Among Medical Students

Characteristic	Student, No.	Exhaustion			Disengagement		
		Mean (SD)	No. (%)	RR (95% CI)	Mean (SD)	No. (%)	RR (95% CI)
Total	114 016	8.2 (3.5)	27 268 (23.9)	NA	12.7 (4.2)	26 292 (23.0)	NA
Marital status							
Single	88 324	8.2 (3.5)	22 028 (24.9)	1 [Reference]	12.8 (4.2)	20 772 (23.5)	1 [Reference]
Married	25 692	8.0 (3.5)	5240 (20.3)	0.81 (0.79-0.84)	12.2 (4.3)	5520 (21.4)	0.91 (0.88-0.93)
Dependent							
None	106 053	8.2 (3.5)	25 733 (24.2)	1 [Reference]	12.7 (4.2)	24 559 (23.1)	1 [Reference]
One or more	7963	8.0 (3.5)	1535 (19.2)	0.79 (0.75-0.83)	11.9 (4.3)	1733 (21.7)	0.93 (0.90-0.98)
Marital status-dependent							
Single, no dependent	87 716	8.2 (3.5)	21 845 (24.9)	1 [Reference]	12.8 (4.2)	20 622 (23.5)	1 [Reference]
Single, dependent	608	8.3 (3.5)	183 (30)	1.20 (1.06-1.37)	13.2 (4.5)	150 (24.6)	1.04 (0.91-1.20)
Married, no dependent	18 337	8.0 (3.5)	3888 (21.2)	0.85 (0.82-0.87)	12.3 (4.2)	3937 (21.4)	0.91 (0.88-0.94)
Married, dependent	7355	8.0 (3.5)	1352 (18.3)	0.73 (0.69-0.78)	11.8 (4.2)	1583 (21.5)	0.91 (0.87-0.95)

Abbreviation: NA, not applicable; RR, relative risk.

Compared with single students without dependents, married students with dependents had the lowest risk of exhaustion (aRR, 0.73; 95% CI, 0.69-0.78), while single students with dependents had an increased risk of exhaustion (aRR, 1.20; 95% CI, 1.06-1.37) and similar risk of disengagement (aRR, 1.04; 95% CI, 0.91-1.20) (Table 1). Compared with single males without dependents, married females (aRR, 0.87; 95% CI, 0.84-0.90) and females with dependents (aRR, 0.71; 95% CI, 0.65-0.78) had lower exhaustion risk (Table 2). Marital status was associated with higher exhaustion risk among females compared with males (21.4% vs 19.6%; interaction-aRR, 1.12; 95% CI, 1.07-1.19; *P* < .001); having dependents had a similar association with exhaustion for both male and female students.

**Table 2.** Association of Intersectional Sex, Marital Status, and Dependent Status With Burnout Among Medical Students

Characteristic	Student, No.	Exhaustion			Disengagement		
		Mean (SD)	No. (%)	RR (95% CI)	Mean (SD)	No. (%)	RR (95% CI)
Total	114 016	8.2 (3.5)	27 268 (23.9)		12.7 (4.2)	26 292 (23.0)	
Male							
Single, no dependent	40 053	8.2 (3.6)	10 158 (25.3)	1 [Reference]	12.8 (4.4)	9480 (23.6)	1 [Reference]
Single, dependent	297	8.2 (3.5)	96 (32.3)	1.27 (1.08-1.49)	13.3 (4.7)	71 (23.9)	1.01 (0.82-1.23)
Married, no dependent	8936	7.9 (3.5)	1804 (20.1)	0.79 (0.76-0.82)	12.1 (4.3)	1910 (21.3)	0.90 (0.86-0.94)
Married, dependent	4925	8.0 (3.5)	910 (18.4)	0.72 (0.67-0.78)	11.7 (4.3)	1076 (21.8)	0.92 (0.87-0.97)
Female							
Single, no dependent	47 663	8.3 (3.4)	11 687 (24.5)	0.96 (0.94-0.98)	12.9 (4.1)	11 142 (23.3)	0.98 (0.96-1.01)
Single, dependent	311	8.4 (3.5)	87 (27.9)	1.10 (0.90-1.34)	13.1 (4.4)	79 (25.4)	1.07 (0.88-1.29)
Married, no dependent	9401	8.1 (3.4)	2084 (22.1)	0.87 (0.84-0.9)	12.5 (4.1)	2027 (21.5)	0.91 (0.87-0.95)
Married, dependent	2430	8.0 (3.4)	442 (18.1)	0.71 (0.65-0.78)	11.9 (4.0)	507 (20.8)	0.88 (0.81-0.95)

Abbreviation: RR, relative risk.

Disengagement risk followed a similar pattern, with reduced risk among married students. No significant increase in disengagement was seen among single students with dependents (Table 2). Marriage and having dependents had similar effect sizes for both male and female students.

## Discussion

In this study, married students and those with dependents had significantly lower exhaustion and disengagement risk compared with their peers. Despite the observed protective associations of marriage and dependent care, trainees report receiving discouragement and insufficient institutional support for family planning.^5^

Our intersectional approach highlights the unique burden of exhaustion. Notably, single students with dependents and married female students reported the highest rates of exhaustion, suggesting that limited social or logistical support that a partner might provide and gendered expectations may reduce the protective effects of marriage and dependent care.^3,6^ Potential strategies to help address these disparities include family planning support, mentorship, and financial assistance.^5^

This study’s cross-sectional design and self-report may be subject to reporting bias and limit causal interpretation. While we used the top quartile as a high burnout risk, future studies should define a clinically meaningful cutoff for student burnout. Despite these limitations, the findings highlight the importance of addressing the challenges and negative perspectives toward family planning that persist despite the protective effects of marriage and dependent care.

## Notes

Supplement.
Data Sharing Statement
