# Omission in Methods

**DOI:** 10.1001/jamanetworkopen.2026.7021

**Published:** 2026-03-24

In the Research Letter titled “Marriage, Dependent Care, and Burnout Among Medical Students,”^1^ published February 16, 2026, information on ethical review of the study was missing from the Methods section. The article has been revised to state: This cross-sectional study used fully deidentified data and was exempt from review per criteria from the New York University Langone Health institutional review board. In addition, a typo in the title of Table 1 was fixed. This article has been corrected.^1^
