# Supplementary material for: Marriage, Dependent Care, and Burnout Among Medical Students
Source: JAMA Netw Open. 2026 Feb 16;9(2):e2559889. doi: 10.1001/jamanetworkopen.2025.59889 (PMC12910393; doi:10.1001/jamanetworkopen.2025.59889)
Supplement: Supplement. — Data Sharing Statement [file jamanetwopen-e2559889-s001.pdf]

Data Sharing Statement

Nguyen. Marriage, Dependent Care, and Burnout Among Medical Students. *JAMA Netw Open*. Published February 16, 2026. doi:10.1001/jamanetworkopen.2025.59889

Data
Data available: No
